# Regulatory effects of cAMP receptor protein (CRP) on porin genes and its own gene in *Yersinia pestis*

**DOI:** 10.1186/1471-2180-11-40

**Published:** 2011-02-23

**Authors:** He Gao, Yiquan Zhang, Lin Yang, Xia Liu, Zhaobiao Guo, Yafang Tan, Yanping Han, Xinxiang Huang, Dongsheng Zhou, Ruifu Yang

**Affiliations:** 1 State Key Laboratory of Pathogen and Biosecurity, Beijing Institute of Microbiology and Epidemiology, Beijing 100071, PR China; 2 Department of Biochemistry and Molecular Biology, Jiangsu University School of Medical Technology, Zhenjiang, Jiangsu 212013, PR China

## Abstract

**Background:**

The cAMP receptor protein (CRP) is a global bacterial regulator that controls many target genes. The CRP-cAMP complex regulates the *ompR-envZ *operon in *E. coli *directly, involving both positive and negative regulations of multiple target promoters; further, it controls the production of porins indirectly through its direct action on *ompR-envZ*. Auto-regulation of CRP has also been established in *E. coli*. However, the regulation of porin genes and its own gene by CRP remains unclear in *Y. pestis*.

**Results:**

*Y. pestis *employs a distinct mechanism indicating that CRP has no regulatory effect on the *ompR-envZ *operon; however, it stimulates *ompC *and *ompF *directly, while repressing *ompX*. No transcriptional regulatory association between CRP and its own gene can be detected in *Y. pestis*, which is also in contrast to the fact that CRP acts as both repressor and activator for its own gene in *E. coli*. It is likely that *Y. pestis *OmpR and CRP respectively sense different signals (medium osmolarity, and cellular cAMP levels) to regulate porin genes independently.

**Conclusion:**

Although the CRP of *Y. pestis *shows a very high homology to that of *E. coli*, and the consensus DNA sequence recognized by CRP is shared by the two bacteria, the *Y. pestis *CRP can recognize the promoters of *ompC*, *F*, and *X *directly rather than that of its own gene, which is different from the relevant regulatory circuit of *E. coli*. Data presented here indicate a remarkable remodeling of the CRP-mediated regulation of porin genes and of its own one between these two bacteria.

## Background

The two major porins of *Escherichia coli*, namely OmpF and OmpC, form non-specific transport channels and allow for the passive diffusion of small, polar molecules (such as water, ions, amino acids, and other nutrients, as well as waste products) across the cell membrane. High and low levels of OmpF and OmpC are respectively expressed at low osmolarities in *E. coli*; as the medium osmolarity increases, OmpF expression is repressed, while OmpC is activated [1,2]. OmpF forms a larger pore (hence a faster flux) than OmpC [3]. OmpC expression is favored when the enteric bacteria, such as *E. coli*, live in the mammalian gut where a high osmolarity (300 mM of NaCl or higher) is observed; in addition, the smaller pore size of OmpC can aid in the exclusion of harmful molecules in the gut. OmpF can predominate in the aqueous habitats, and its larger pore size can assist in scavenging for scarce nutrients from the external aqueous environments.

OmpX represents the smallest known channel protein. OmpX expression in *Enterobacter *is inducible under high osmolarity, which is accompanied by the repressed expressions of OmpF and OmpC [4-6]. The over-expression of OmpX can balance the decreased expression of non-specific porins, OmpF and OmpC, for the exclusion of small harmful molecules. However, whether or not OmpX functions as a porin to modulate the membrane permeability is still unclear.

**Figure 1:**
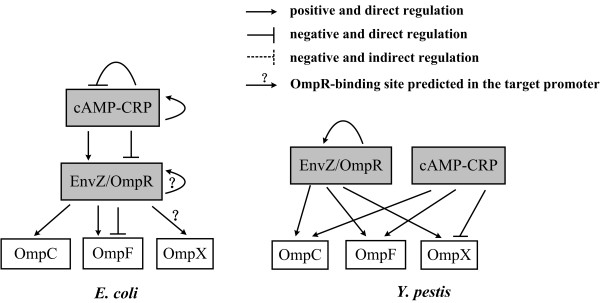
**Comparison of porin regulation by OmpR and CRP in *E. coli *and *Y. pestis***. The OmpR-mediated reciprocal regulation of OmpF and OmpC in *E. coli *was discussed in the text [2,7,8]. In addition, CRP controlled the production of porins indirectly through its direct regulation of OmpR/EnvZ in *E. coli *[8,15]. As shown in this study, *Y. pestis *employs a distinct mechanism indicating that CRP has no regulatory effect on the *ompR-envZ *operon, although it stimulates *ompC *and *ompF *directly, while repressing *ompX *at the same time. It is likely that OmpR and CRP respectively sense different signals, medium osmolarity, and cellular cAMP levels to regulate porin genes independently. As shown previously [12], *Y. pestis *OmpR simulates *ompC*, *F*, *X*, and *R *directly by occupying the target promoter regions. Notably, all of *ompF*, *C*, *X*, and *R *give a persistent and dramatic up-regulation with the increasing medium osmolarity in *Y. pestis*, which is dependent of OmpR. Upon the shifting of medium osmolarity, porin expression in *Y. pestis *is contrary to the reciprocal regulation of OmpF and OmpC in *E. coli*. The F1-F2-F3 and C1-C2-C3 sites are detected for *ompF *and *ompC *of *Y. pestis*, respectively. Remarkably, the F4 site is absent from the upstream region of *ompF*, which probably destroys the OmpR-mediated blocking mechanism of *ompF *at high osmolarity. In *E. coli*, CRP acts as both repressor and activator for its own gene [28,29]. However, no transcriptional regulatory association between CRP and its own gene was detected in *Y. pestis*.

The osmosensor histidine protein kinase EnvZ can phosphorylate the response regulator OmpR, which constitutes a two-component signal transduction and regulatory system. The reciprocal regulation of OmpF and OmpC in *E. coli *is mediated by phosphorylated OmpR (OmpR-P) [2,7,8] (Figure 1). OmpR-P binds to four (F4, F1, F2, and F3 from the 5' to 3' direction) and three (C1, C2, and C3) sites within the upstream regions of *ompF *and *ompC*, respectively, with each containing two tandem 10 bp subsites ('a' and 'b') bound by two OmpR-P molecules. At low osmolarity, OmpR-P tandemly binds to F1 and F2 (and somewhat loosely to F3) in order to activate the transcription of *ompF*; meanwhile OmpR-P occupies C1 but not C2 and C3, which is not sufficient to stimulate the transcription of *ompC*. With increasing osmolarity, the cellular levels of OmpR-P elevate, and OmpR-P binds to C2 and C3 cooperatively, allowing for the transcription of *ompC*. At high osmolarity, OmpR-P is also capable of binding to F4, which is a weak site upstream F1-F2-F3. Due to the tandem binding of OmpR-P on F4 and F1-F2-F3, the upstream DNA of *ompF *forms a circular loop, effectively blocking the *ompF *transcription.

OmpR contributes to the building of resistance against phagocytosis and survival within macrophages, which is likely conserved in all the pathogenic yersiniae, namely, *Y. enterocolitica *[9,10], *Y. pseudotuberculosis *[11], and *Y. pestis *[12]. However, in contrast to *Y. enterocolitica *and *Y. pseudotuberculosis*, the virulence of *Y. pestis *is likely unaffected by the *ompR *null mutation. *Y. pestis *OmpR directly regulates *ompC*, *F*, *X*, and *R *through OmpR-promoter DNA association (Figure 1). High osmolarity induces the transcription of all the porin genes (*ompF*, *C*, and *X*) in *Y. pestis*, in contrast with their reciprocal regulation in *E. coli*. The major difference is that *ompF *transcription is not repressed at high osmolarity in *Y. pestis*, which is likely due to the absence of a promoter-distal OmpR-binding site for *ompF*.

cAMP Receptor Protein (CRP) is a global regulator, which controls a large array of target genes [13,14]. CRP binds to its sole cofactor cAMP to form the CRP-cAMP complex for binding to specific DNA sequence within the target promoters [13]. CRP-cAMP activates transcription by binding to specific sites, often upstream of the core promoter (-10 and -35 elements), where it directly interacts with RNA polymerase; it also represses the expression of a few genes where the binding site overlaps with or downstream the core promoter. The CRP-cAMP consensus binding site is TGTGA-N6-TCACA, and variations on this consensus sequence can influence the affinity of CRP-cAMP to bind to different sites, resulting in the regulation of different operons by CRP-cAMP. Meanwhile, cAMP is synthesized from ATP by adenylyl cyclase encoded by *cyaA*. CRP-cAMP regulates the *ompR-envZ *operon in *E. coli *directly, involving both positive and negative regulation of multiple *ompR-envZ *promoters [15]. On the other hand, it controls the production of porins indirectly through its direct regulation of EnvZ/OmpR in *E. coli *(Figure 1).

CRP is a virulence-required regulator of several bacterial pathogens, including *Y. pestis *[16,17]. The *crp *disruption in *Y. pestis *leads to a much greater loss of virulence by subcutaneous infection relative to intravenous inoculation [16]. CRP directly stimulates the expression of plasminogen activator [16,18], a key virulence factor essential for bubonic and primary pneumonic plague [19,20], while directly repressing the *sycO-ypkA-yopJ *operon encoding the chaperone SycO and the effectors YpkA and YopJ of the plasmid pCD1-borne type III secretion system [21].

This study discloses that *Y. pestis *employs a distinct mechanism indicating that CRP has no regulatory effect on the *ompR-envZ *operon, although it stimulates *ompC *and *ompF *directly, while repressing *ompX *at the same time (Figure 1). In addition, no transcriptional regulatory association between CRP and its own gene could be detected in *Y. pestis*, which is also related to the fact that CRP acted as both repressor and activator for its own gene in *E. coli*. It is likely that *Y. pestis *OmpR and CRP respectively sensed different signals, namely medium osmolarity, and cellular cAMP levels, to regulate porin genes independently.

## Methods

### Bacterial strains

The wild-type (WT) *Y. pestis *biovar microtus strain 201 is avirulent to humans but highly lethal to mice [22]. The base pairs 43 to 666 of *ompR *(720 bp in total length) or the entire region of *crp *was replaced by the kanamycin resistance cassette, to generate the *Y. pestis ompR *and *crp *null mutants. These mutants were designated as *ΔompR *[12] and *Δcrp *[16,21], respectively. All the DNA sequences mentioned in this study were derived from the genomic data of CO92 [23]. The construction of the complemented mutant strain *C-crp *was also described in a previous work [16]. All the primers used in this study, which were designed using the Array Designer 3.0 or Primer Premier 5.0 software, were listed in Additional File 1.

### Bacterial growth and RNA isolation

Overnight cultures (an OD_620 _of about 1.0) of WT, *Δcrp *or *ΔompR *in the chemically defined TMH medium [24] were diluted into the fresh TMH with a 1:20 ratio. Bacterial cells were grown at 26°C to the middle exponential growth phase (an OD_620 _of about 1.0). To trigger the high osmolarity conditions in OmpR-related experiments, a final concentration of 0.5 M sorbitol was added [25], after which the cell cultures were allowed to grow for an additional 20 min. For all CRP-related *in vivo *experiments, 1 mM cAMP acting as the activator of CRP was added into the fresh TMH medium [16,21].

Total RNA from bacterial cells was extracted using the TRIzol Reagent (Invitrogen) without DNA removing step (for RT-PCR and primer extension) or by using MasterPure™RNA Purification kit (Epicenter) with the removal of contaminated DNA (for microarray) [16,21]. Immediately before harvesting, bacterial cultures were mixed with RNAprotect Bacteria Reagent (Qiagen) to minimize RNA degradation. RNA quality was monitored by agarose gel electrophoresis, and RNA quantity was determined using a spectrophotometer.

### Quantitative RT-PCR

Gene-specific primers were designed to produce a 150 to 200 bp amplicon for each gene. The contaminated DNA in RNA samples was removed using the Amibion's DNA-free™Kit. cDNAs were generated using 5 μg of RNA and 3 μg of random hexamer primers. Using 3 independent cultures and RNA preparations, quantitative RT-PCR was performed in triplicate as described previously through the LightCycler system (Roche) together with the SYBR Green master mix [16,21]. The PCR reaction mixture contained 2 μl of 10× PCRbuffer, 2 μl of 25 mmol/l MgCl_2_, 0.4 μl of 5 U/μl ExTaq DNA polymerase (Takala), 1 μl of 1:500 SYBR Green I, 0.3 μl of each primer (10 μmol/l), 0.16 μl of 10 mmol/l dNTP, and 2 μl of cDNA templates, with the addition of H_2_O to arrive at a total volume of 20 μl. After pre-denaturation at 95°C for 3 min at a temperature transition rate of 20°C/s, PCR amplification was conducted at 45 cycles of denaturation at 95°C for 2 s at 20°C/s, annealing at 58°C for 4 s at 20°C/s and extension at 72°C for 8 s at 20°C/s, after which a single fluorescence measurement was taken at the end of the extension step. After amplification, a final melting curve was recorded by heating to 95°C, cooling to 65°C at 20°C/s, followed by a 60 s holding period at 65°C before heating slowly at 0.2°C/sec to 95°C. On the basis of the standard curves of 16 S rRNA expression, the relative mRNA level was determined by calculating the threshold cycle (ΔCt) of each gene using the classic ΔCt method. Negative controls were performed using 'cDNA' generated without reverse transcriptase as templates. Reactions containing primer pairs without template were also included as blank controls. The 16 S rRNA gene was used as an internal control to normalize all the other genes [16]. The transcriptional variation between the WT and mutant strain was calculated for each gene. A mean ratio of two was taken as the cutoff of statistical significance.

### Primer extension assay

For the primer extension assay [16,21], about 10 μg of total RNA from each strain was annealed with 1 pmol of [γ-^32^P] end-labeled reverse primer. The extended reverse transcripts were generated as described in the protocol for Primer Extension System-AMV Reverse Transcriptase (Promega). The yield of each primer extension product would indicate the mRNA expression level of the corresponding gene in the corresponding strain, and further could be employed to map the 5' terminus of RNA transcript for each gene. The same labeled primer was also used for sequencing with the fmol^® ^DNA Cycle Sequencing System (Promega). The primer extension products and sequencing materials were concentrated and analyzed using 8 M urea-6% polyacrylamide gel electrophoresis. The result was detected by autoradiography (Kodak film).

### LacZ reporter fusion and β-Galactosidase assay

The 500 to 600 bp upstream DNA region of each indicated gene (Table 1) was obtained by PCR with the ExTaq™ DNA polymerase (Takara) using *Y. pestis *201 genome DNA as the template. PCR fragments were then cloned directionally into the *Eco*RI and *Bam*HI sites of plasmid pRW50 that harbors a tetracycline resistance gene and a promotorless *lacZ *reporter gene [26]. Correct cloning was verified by DNA sequencing. *Y. pestis *was transformed with the recombinant plasmids and grown as described in microarray analysis. The empty plasmid pRW50 was also introduced into both strains as negative control. β-Galactosidase activity was measured on cellular extracts using the β-Galactosidase Enzyme Assay System (Promega) [16,21]. Assays were performed in triplicate. A mean value of two-fold change was taken as the cutoff of statistical significance.

**Table 1:** Genes tested in both computational and biochemical assays

Gene ID	Gene	Regulation	Computational matching of regulatory consensus			Position of DNA fragment used ^§^	
				
			Position§	Sequence	Score	LacZ	Footprinting
YPO1222	*ompC*	+	R-191...-169	AAACAGTGAGTTATAGCACATAT	12.3	-379...+130	-281...-26
YPO1411	*ompF*	+	D-131...-109	ACTTTGTGACTTAGATCGAATTT	10.73	-328...+143	-237...-4
YPO2506	*ompX*	-	D-156...-134	AGTATGTGACCTCCATCACCCAA	11.68	-374...+123	-321...+4
YPO0136	*ompR*	NO	-	-	0	-409...+83	-409...+83
YPO0175	*crp*	NO	R+235...+257	GAACTCTGAGCCCTGTTAAGTTA	1.44	-147...+344	-147...+344

§, The numbers indicate the nucleotide positions upstream of the transcription start sites

+, positive and direct regulation

-, negative and direct regulation

### Preparation of His-OmpR and His-CRP proteins

The entire coding region of *ompR *or *crp *was amplified from *Y. pestis *201 and then cloned directionally into the *Bam*HI and *Hind*III sites of plasmid pET28a, which was verified by DNA sequencing [16,21]. The recombinant plasmid encoding a His-protein was transformed into BL21λDE3 cells. Over-expression of His-OmpR or His-CRP in the LB medium was induced by adding 1 mM IPTG (isopropyl-b-D-thiogalactoside). The over-expressed proteins were purified under native conditions with nickel loaded HiTrap Chelating Sepharose columns (Amersham). The purified and eluted proteins were concentrated to a final concentration of 0.1 to 0.3 mg/ml with the Amicon Ultra-15 (Millipore), which was confirmed by SDS-PAGE for purity. The purified proteins were stored at -80°C until further use.

### DNase I footprinting

The promoter DNA region (Table 1) was prepared by PCR amplification performed with the promoter-specific primer pairs, including a 5'-^32^P-labeled primer (either forward or reverse) and its non-labeled counterpart. The PCR products were purified using QiaQuick cleanup columns (Qiagen). Increasing amounts of purified His-protein were incubated with the labeled DNA fragment (2 to 5 pmol) for 30 min at room temperature in a binding buffer containing 10 mM Tris-Cl (pH7.4), 50 mM KCl, 0.5 mM DTT, 1 mM MgCl_2_, 4% glycerol, 0.05 mg/ml BSA, 0.05 mg/ml shared salmon sperm DNA and 0.5 mM EDTA, with a final volume of 10 μl [16,21]. To achieve the OmpR phosphorylation, 25 mM fresh acetyl phosphate was added in the binding buffer and incubated with purified His-OmpR for 30 min, after which the labeled DNA was added for additional incubation for 30 min. To activate CRP, 2 mM cAMP was mixed with purified His-CRP in the DNA-binding reactions. To initiate DNA digestion, 10 μl of Ca^2+^/Mg^2+ ^solution (5 mM CaCl_2 _and 10 mM MgCl_2_) was added, followed by incubation for 1 min at room temperature. Afterwards, the optimized RQ1 RNase-Free DNase I (Promega) was added to the reaction mixture, and the mixture was incubated at room temperature for 30 to 90 s. The cleavage reaction was stopped by adding 9 μl of the stop solution (200 mM NaCl, 30 mM EDTA, and 1% SDS) followed by DNA extraction and precipitation. The partially digested DNA samples were then analyzed in a 6% polyacrylamide/8 M urea gel. Protected regions were identified by comparing these with the sequence ladders. For sequencing, the fmol^® ^DNA Cycle Sequencing System (Promega) was used, and the final result was detected by autoradiography (Kodak film).

### Computational promoter analysis

The 300 bp promoter regions upstream of the start codon of each indicated gene was retrieved using the '*retrieve-seq*' program [27]. The '*matrices-paster' *tool [27] was used to match the relevant position-specific scoring matrix (PSSM) within the above promoter regions.

## Results

### *Non-polar mutation of *ompR *or *crp

The *ompR *and *crp *null mutants designated as *ΔompR *and *Δcrp*, respectively, have been evaluated in the present study. Non-polar mutation of *ompR *has been confirmed previously with the complemented *ompR *mutant [12]. To prove the non-polar mutation of *crp*, we constructed the pRW50-harboring fusion promoter, which consisted of a promoter-proximal region of *ompF *and promoterless *lacZ*, and then transformed into WT, *Δcrp *and *C-crp *(the complemented *crp *mutant), respectively (Additional file 2). The *ompF *gene was positively regulated by CRP as determined by several distinct methods (see below). As expected, the *ompF *promoter activity (β-galactosidase activity) decreased significantly in *Δcrp *relative to WT grown in the TMH medium with the addition of 1 mM cAMP, but showed almost no difference between WT and C-*crp*.

### *Direct regulation of *ompC, F *and *X *by CRP*

**Figure 2:**
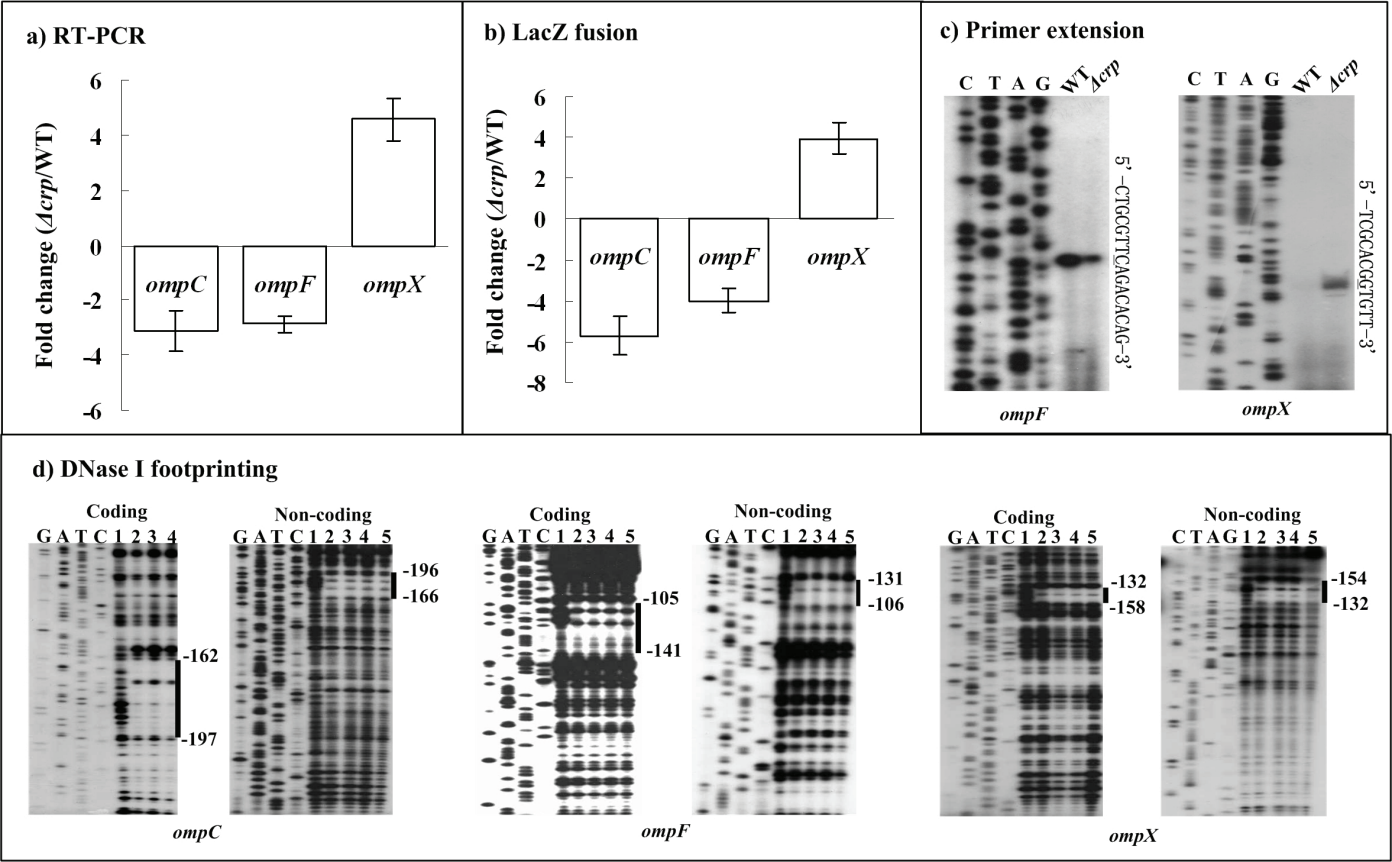
**Regulation of *ompC*, *F *and *X *by CRP**. **a)** Quantitative RT-PCR. The mRNA levels of each indicated gene were compared between *Δcrp *and WT. This figure shows the increased (positive number) or decreased (minus one) mean fold for each gene in *Δcrp *relative to WT. **b)** LacZ fusion reporter. A promoter-proximal region of each indicated gene was cloned into pRW50 containing a promotorless *lacZ *reporter gene, and transformed into WT or *Δcrp *to determine the promoter activity (β-Galactosidase activity in cellular extracts). The empty plasmid was also introduced into the corresponding strain as negative control, which gave extremely low promoter activity (data not shown). β-Galactosidase activity in each tested cellular extract was subtracted with that of negative control. This figure shows the increased (positive number) or decreased (minus one) mean fold for the detecting promoter activity in *Δcrp *relative to WT. **c)** Primer extension. Primer extension assays were performed for each indicated gene using total RNAs isolated from the exponential-phase of WT or *Δcrp*. An oligonucleotide primer complementary to the RNA transcript of each gene was designed from a suitable position. The primer extension products were analyzed with 8 M urea-6% acrylamide sequencing gel; lanes C, T, A, and G represent the Sanger sequencing reactions, respectively. On the right side, DNA sequences are shown from the bottom (5') to the top (3'), and the transcription start sites were underlined. **d)** DNase I footprinting. The labeled DNA probe was incubated with various amounts of purified His-CRP (lanes 1, 2, 3, 4, and 5 containing 0, 5, 10, 15 and 20 pmol, respectively) in the presence of 2 mM cAMP, and subjected to DNase I footprinting assay; lanes G, A, T, and C represent the Sanger sequencing reactions, respectively. The protected regions (bold lines) are indicated on the right-hand side. The numbers indicated the nucleotide positions upstream the transcriptional start sites.

The quantitative RT-PCR analysis was also performed to compare the mRNA levels of each gene tested in *Δcrp *and WT in the presence of 1 mM cAMP. Both RT-PCR (Figure 2a) and *lacZ *fusion reporter (Figure 2b) assays revealed that the expression of *ompC *or *F *decreased significantly in *Δcrp *relative to WT, while that of *ompX *increased.

In addition, primer extension experiments (Figure 1c) were conducted for *ompC*, *F*, and *X *to detect the yield of primer extension product that represented the relative activity of each target promoter in *Δcrp *or WT. A single promoter was transcribed for *ompF *or *X*, which was dependent on CRP. No primer extension product could be detected for *ompC *in both *ΔompR *and WT after repeated efforts, which might be due to the limitation of the primer extension assay. Meanwhile, the transcriptional levels of *ompF *or *X *in *ΔompR *and WT, determined by primer extension experiments herein (Figure 1c), was consistent with the RT-PCR and *lacZ *fusion reporter data.

A previously described CRP consensus (PSSM) of *Y. pestis *[16] was used to scan the 300bp upstream DNA regions of *ompC*, *F *and *X*, with a cutoff score value of 7; CRP consensus-like sequences were also predicted for *ompC*, *F*, and *X *(Table 1). As determined by DNase I footprinting (Figure 2d), a purified His-CRP protein in the presence of 2 mM cAMP protected a single distinct region upstream of each target gene against DNase I digestion in a dose-dependent pattern.

Taken together, CRP-cAMP stimulated *ompC *and *ompF*, while repressing *ompX *through the CRP-promoter DNA association in *Y. pestis*.

### No autoregulation of CRP

**Figure 3:**
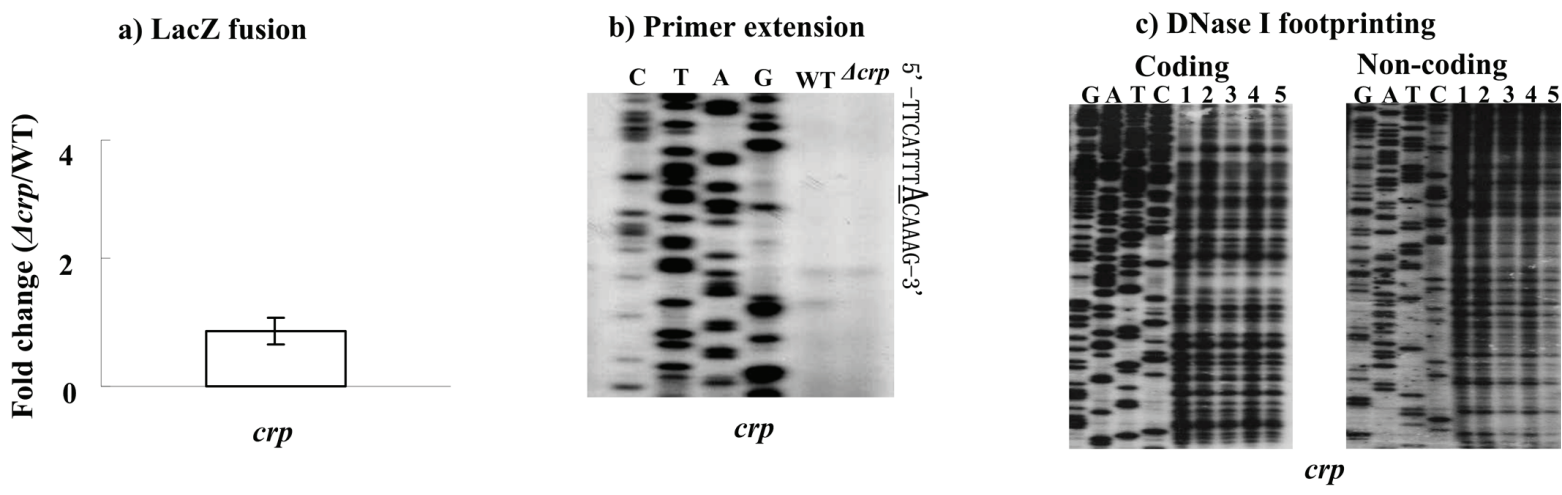
**No autoregulation of CRP**. **a)** LacZ fusion reporter. A promoter-proximal region of *crp *was cloned into pRW50 and transformed into WT or *Δcrp *to determine their promoter activities, respectively. This figure shows the increased mean fold for the activity in *Δcrp *relative to WT. **b)** Primer extension. Primer extension assay was performed for *crp *using total RNAs from WT or *Δcrp*. On the right side, DNA sequences are shown from the bottom (5') to the top (3'), and the transcription start sites are underlined. **c) **DNase I footprinting. The labeled upstream DNA fragment of *crp *was incubated with 0, 5, 10, 15, and 20 pmol of purified His-CRP in lanes 1 to 5, respectively, in the presence of 2 mM cAMP. No footprint region was detected.

Both *lacZ *fusion reporter (Figure 3a) and primer extension (Figure 3b) assays showed almost the same levels of *crp *expression in both WT and *Δcrp; *moreover, the footprinting analysis (Figure 3c) indicated no direct association between His-CRP and *crp *promoter region in the presence 2 mM cAMP. Thus, no transcriptional auto-regulation of CRP could be detected in *Y. pestis *under the growth conditions used in this work.

### No regulatory interaction between OmpR and CRP

**Figure 4:**
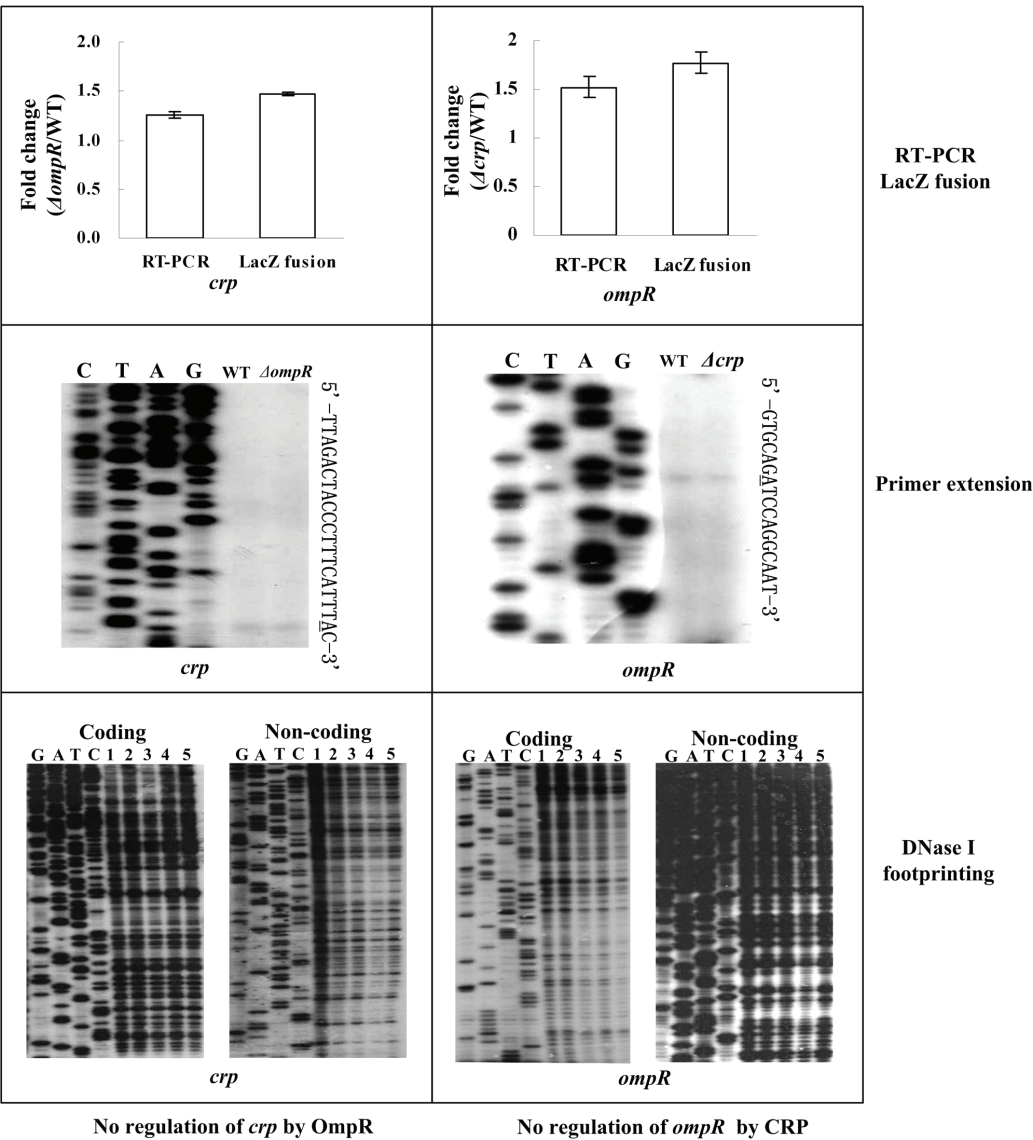
**No regulatory interaction between OmpR and CRP**. For RT-PCR and LacZ fusion experiments, we show the mean fold increase of the mRNA level (RT-PCR) or the detecting promoter activity (LacZ fusion) for *crp *or *ompR *in *ΔompR *or *Δcrp *relative to WT. For primer extension experiments, we show the primer extension product for *crp *or *ompR *in WT or *Δcrp *or *ΔompR*, and DNA sequences on the right side from the bottom (5') to the top (3'); the transcription start sites are underlined. For DNase I footprinting experiments, the labeled DNA probe of *crp *or *ompR *was incubated with 0, 5, 10, 15, and 20 pmol of purified His-CRP (with addition of 2 mM cAMP) or His-OmpR (in the presence of 25 mM acetyl phosphate) in lanes 1 to 5, respectively. No footprint region was detected.

As determined by both primer extension and *lacZ *fusion reporter assays, the *ompR *gene was expressed at almost the same level in both WT and *Δcrp*; likewise, no difference in the *crp *expression was observed between WT and *ΔompR *(Figure 4). Moreover, the footprinting analysis indicated no direct association between the His-CRP protein and the *ompR *promoter region or between the His-OmpR-P protein and the *crp *promoter region (Figure 4). Accordingly, under the growth conditions used in this work, OmpR had no regulatory effect on *crp*, and in turn, CRP did not regulate *ompR*.

### Structure of promoter-proximal regions

**Figure 5:**
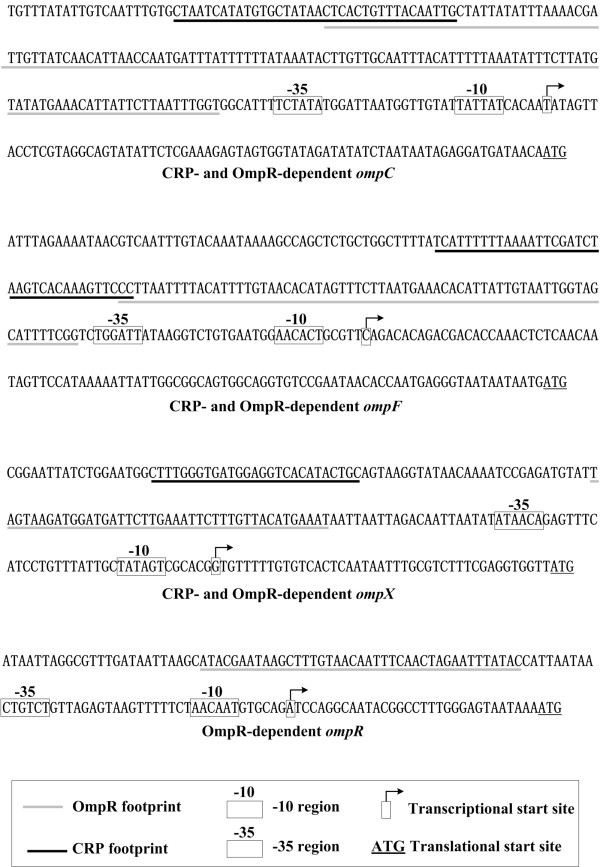
**Promoter structure for *ompC*, *F*, *X *and *R***. The start codon (ATG) of each gene is shown at the 3' terminus. The nucleotide number corresponding to the transcription start site was taken as "+1", from which the promoter -10 and-35 elements were predicted accordingly. Data of OmpR-promoter DNA association came from the previous data [12].

The footprint regions determined by DNase I footprinting were considered as the binding sites of relevant regulators. The primer extension product could be used to map the 5' terminus of RNA transcript of each gene tested, allowing for the determination of transcriptional start sites and localization of the core promoter region (-10 and -35 elements). Considering the data here and those described previously [12], we depicted OmpR- or CRP-binding sites, transcriptional start sites, and -10/-35 elements within the promoter-proximal regions of *ompC*, *F*, *X *and *R *(Figure 5), resulting in a map of regulator-promoter DNA association for mediating transcriptional regulation. Since we failed to detect the 5' terminus of the RNA transcript for *ompC *using primer extension assay, a transcriptional start site was predicted for this gene with the *NNPP *tool http://searchlauncher.bcm.tmc.edu/seq-search/gene-search.html. The results showed that a single distinct promoter was transcribed for all the four genes, and the detecting promoters for *ompC*, *F*, and *X *were dependent on both OmpR and CRP, while that of *ompR *was regulated by its own protein product but not by CRP. A single distinct OmpR- or CRP-binding site was respectively detected in *ompC*, *F*, and *X*, all of which were upstream of the promoter -35 elements. The detecting OmpR- and CRP-binding sites contained the corresponding consensus-like sequences as predicted by computational promoter analysis.

### No interplay of OmpR and CRP at target promoters

**Figure 6:**
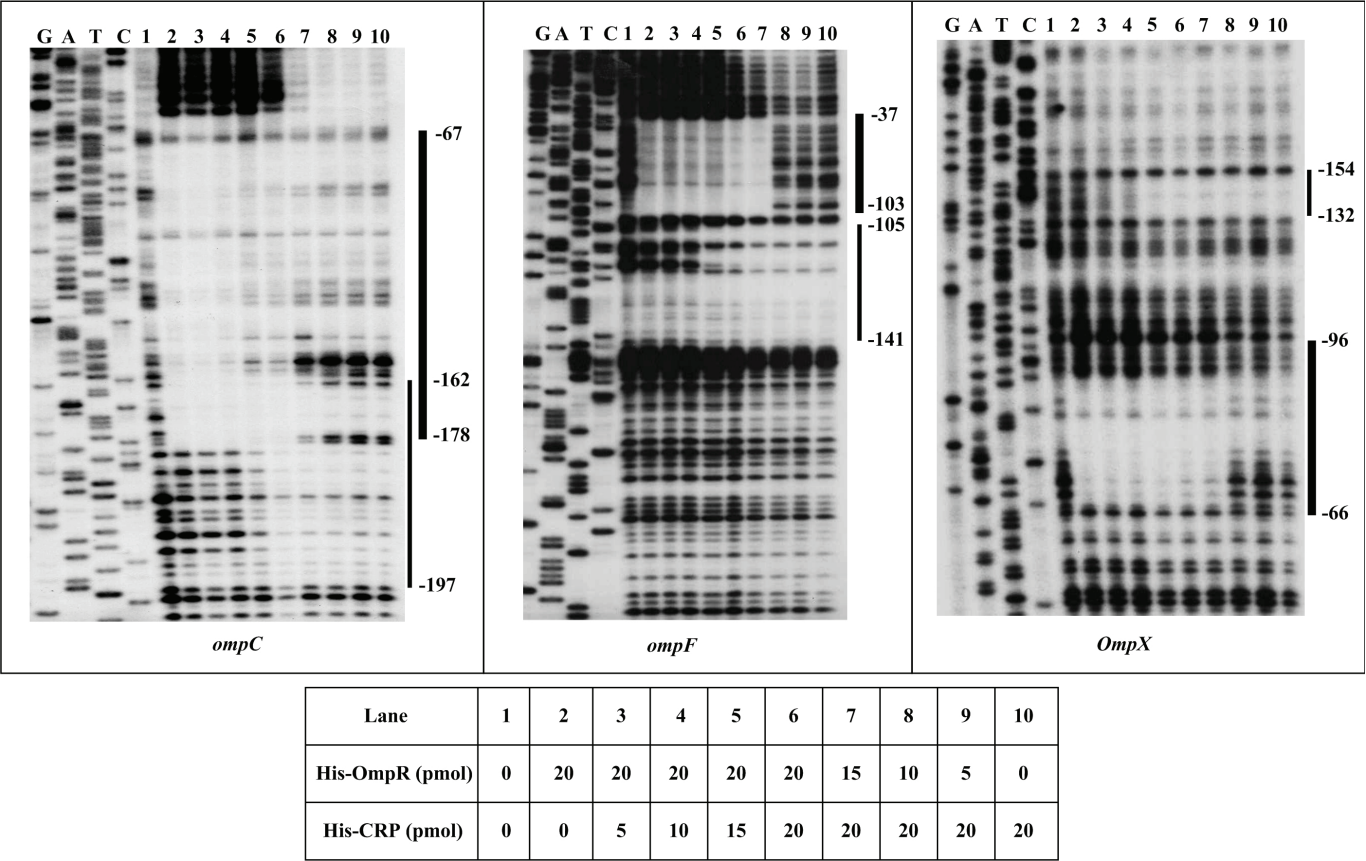
**Competitive DNase I footprinting analysis**. The labeled coding strand of the promoter-proximal DNA fragment of each indicated gene was incubated with His-OmpR, His-CRP or both in the presence of acetyl phosphate and cAMP for DNase I footprinting assay. The thick bold lines indicate the OmpR protected regions, while the thin ones denote CRP footprints. The numbers also indicate the nucleotide positions upstream the transcriptional start sites. We also show the amounts of His-OmpR and His-CRP used in each lane.

There was no overlapping of OmpR- and CRP-binding sites for *ompX*; however, overlapping regions that were 17 and 2 bp in length were observed for *ompC *and *ompF*, respectively. We performed further footprinting experiments using the coding strands of the promoter-proximal DNA fragments of *ompC*, *F*, and *X *with different amounts of OmpR and CRP in various reactions (Figure 6). His-CRP protected each promoter region tested in a dose-dependent manner when His-OmpR-P was at the highest amount (20 pmol), and vice versa. Both His-CRP and His-OmpR-P at the highest amounts were able to bind together to each promoter region tested. These results indicated that no competitive binding occurred between them to these target promoters. It was likely that OmpR and CRP sensed different signals to regulate *ompC*, *F*, and *X *in an independent manner.

## Discussion

### Autoregulation of CRP-cAMP

In *E. coli*, CRP acts as both repressor and activator for its own gene [28,29], while also repressing the *cyaA *expression [30]. Enteric bacteria catabolize other sugars only when the supply of glucose has become depleted, whereas the presence of glucose prevents the bacteria from catabolizing alternative sugars, which is referred to as catabolite repression mainly mediated by CRP-cAMP for positively controlling the metabolism of alternative sugars [13,14]. A mode for the regulation of the CRP-cAMP machinery during catabolite repression could be established in *E. coli *as follows [28,29,31,32]: i) the presence of glucose (catabolite repression) reduces the cAMP level by decreasing the phosphorylated form of enzyme IIAGlc, which is involved in the activation of CyaA, after which the reduction of cAMP can affect the positive autoregulatory mechanism of *crp *(see below) to cause a further decrease of *crp *expression; and ii) once at cAMP-rich conditions (e.g., the replacement of glucose by mannitol), CRP-cAMP activates the *crp *transcription by occupying the CRP binding site II, after whichthe elevated expression of CRP-cAMP enables its recognition of the CRP binding site I located 40 bp downstream the *crp *transcription start site (thereby preventing the occupation of RNA polymerase at the *crp *promoter), while repressing the *cyaA *transcription; and finally, a return to basal levels of CRP and cAMP is induced.

It is noteworthy that transcriptional regulatory association between CRP and its own gene can be detected in *Y. pestis*. However, CRP bound to a DNA region that overlapped the promoter -10 region of *cyaA*, can block the entry of the RNA polymerase for repressing the transcription of *cyaA *in *Y. pestis *(data unpublished). Since the *cyaA*-encoding adenylyl cyclase is a key enzyme catalyzing the synthesis of cAMP, which is the sole essential cofactor of CRP, repression of cAMP production by CRP represents a mechanism for negative modulation of cellular CRP function.

### CRP-cAMP and osmoregulation

The cellular cAMP levels are significantly increased at high osmolarity relative to low osmolarity in *E. coli*; this osmoregulation requires the cAMP molecule, and is mainly exerted at the transcriptional level although the control at the posttranscriptional level cannot be excluded [33]. The replacement of glucose by other catabolites in the medium triggers the elevation of both cAMP and CRP levels in *E. coli *[32,34], resulting in the increase and decrease of OmpF and OmpC levels, respectively [8]. OmpF allows a higher number of compounds to enter the cell than the more restrictive OmpC channel, thereby contributing to the transport of amino acids as a secondary carbon/energy source for *E. coli *in the absence of glucose [15]. CRP-cAMP directly regulates the *ompR-envZ *operon in *E. coli *through the process of binding to a single site within the upstream region of *ompR *[15]. Four transcripts were detected for the *ompR-envZ *operon, while CRP-cAMP negatively regulates the two promoters that overlap the CRP binding site and is positive for the other two that are located further downstream from this site [15]. Thus, CRP-cAMP controls the production of porins indirectly through its direct action on *ompR-envZ *in *E. coli*. In contrast, *Y. pestis *has evolved a distinct mechanism, wherein CRP-cAMP has no regulatory effect on the *ompR-envZ *operon; rather, consistent with the findings reported here, it directly stimulates *ompC *and *ompF*, while repressesing *ompX*. Regulation of *ompX *by CRP through the CyaR small RNA has been established in both *Salmonella enterica *[35] and *E. coli *[36,37]; the CRP-cAMP complex is a direct activator of the transcription of CyaR, which further promotes the decay of the *ompX *mRNA, under conditions in which the cAMP levels are high. Transcription of the P1 promoter of the *E. coli proP *gene, which encodes a transporter of osmoprotectants (proline, glycine betaine, and other osmoprotecting compounds) is strongly induced by a shift from low to high osmolarity conditions [38,39]. CRP-cAMP functions as an osmosensitive repressor of the *proP *P1 transcription through CRP-cAMP-promoter DNA association [38,39]. The *proP *P2 promoter is induced upon entry into the stationary phase to protect cells from osmotic shock; the CRP-cAMP and Fis regulators synergistically coactivate the P2 promoter activity, through independently binding to two distinct P2 promoter-proximal regions and making contacts with the two different C-terminal domains of the a subunit of RNA polymerase [40]. These findings suggest that CRP-cAMP functions in certain contexts in osmoregulation of gene expression, in addition to its role in catabolite control.

### Remodeling of regulatory circuits of porin genes

The evolutionary remodeling of regulatory circuits can bring about phenotypic differences between related organisms [41]. This is of particular significance in bacteria due to the widespread effects of horizontal gene transfer. A set of newly acquired virulence genes (e.g., *pla *and the pH6 antigen genes) in *Y. pestis *has evolved to integrate themselves into the 'ancestral' CRP or RovA regulatory cascade [16,18,42]. The PhoP regulons have been extensively compared in *Y. pestis *and *S. enterica *[41,43]. The orthologous PhoP proteins in these bacteria differ both in terms of their ability to promote transcription and in their role as virulence regulators. The core regulon controls the levels of active PhoP protein and mediates the adaptation to low Mg^2+ ^conditions. In contrast, the variable regulon members contribute species-specific traits that allow the bacteria to incorporate newly acquired genes into their ancestral regulatory circuits [41,43]. In general, *Y. pestis *integrates virulence genes acquired laterally to coordinate their expressions within its regulatory backbone cascades to maintain the homeostasis during infection [44].

Data presented herein, as well as those described previously [12], disclose a regulatory circuit involving CRP-cAMP, EnvZ/OmpR, and a set of porins in *Y. pestis *(Figure 1). Noticeable remodeling was observed when this regulatory circuit was compared to the counterpart in *E. coli *(Figure 1). The *Y. pestis *CRP-cAMP or EnvZ/OmpR has shown a very high homology to the orthologous one in *E. coli *(data not shown), and CRP [16] or OmpR [12] from these two bacteria share an identical consensus sequence, indicating that conserved signals recognized by CRP or OmpR are shared by these bacteria. However, the promoter regions of *crp *and *ompR*, *C*, *F*, and *X *have undergone genetic variations between *E. coli *and *Y. pestis*, thereby promoting relevant target genes to split from or integrate into the CRP or OmpR regulon of *Y. pestis *relative to that of *E. coli*. The complex regulatory circuit of porins may contribute to bacterial adaptation to the hosts.

## Conclusion

*Y. pestis *CRP-cAMP has no regulatory effect on the *ompR-envZ *operon, although it stimulates *ompC *and *ompF *directly, while repressesing *ompX *at the same time. This is different from the fact that CRP-cAMP regulates *ompR-envZ *directly in *E. coli *and further controls the porin production indirectly through its direct action on *ompR-envZ*. No transcriptional regulatory association between CRP and its own gene can be detected in *Y. pestis*, which is also in contrast to the observation that CRP acts as both repressor and activator for its own gene in *E. coli*.

## Authors' contributions

DZ and RY conceived the study and designed the experiments. HG and YZ performed all the experiments. LY, XL, and ZG contributed to RT-PCR, primer extension assay and DNA binding assays. ZG and YT participated in protein expression and purification. DZ, XH, and YH performed computational analysis and figure construction. The manuscript was written by DZ and HG, and was revised by RY. All the authors read and approved the final manuscript.

## Supplementary Material

Additional file 1 **Oligonucleotide primers used in this study**. Click here for file

Additional file 2 **Promoter activity of *ompF *within WT, *Δcrp *and *C-crp***. Click here for file
