# Supplementary material for: Regulatory effects of cAMP receptor protein (CRP) on porin genes and its own gene in Yersinia pestis
Source: BMC Microbiol. 2011 Feb 23;11:40. doi: 10.1186/1471-2180-11-40 (PMC3050693; doi:10.1186/1471-2180-11-40)
Supplement: Additional file 1 — Oligonucleotide primers used in this study. [file 1471-2180-11-40-S1.DOC]

**Supplementary Table S1. Oligonucleotide primers used in this study**

| **Gene** | **Primers (5'-3', Forward/Reverse primer)** |
| --- | --- |
| **For RT-PCR** | |
| *ompR* | CAAGATTCTGGTCGTTGATG/ATCAGGTCAAGTACCATCAG |
| *crp* | CTCTCGAATGGTTCCTGTC/ATCATCTCTTTGCCTTCCTC |
| **For gene mutation** | |
| *ompR* | ATGCAAGAGAATCACAAGATTCTGGTCGTTGATGACGATATGTTGTGTCTCAAAATCTCTG/  CCGTCCGGTACAAAGACGTAGCCTAGACCCCACACCGTCTGAAAGCCGCCGTCCCGTCAAG |
| *crp* | TGTTAAGTTAGGCAGCGATAACAACAGAGGATAACAGCGAAGATTGCAGCATTACACG/ CGCTAATAGCTTCAGCTTTAACGCCGGTTTTTAGAGGGAATGTAACGCACTGAGAAGC |
| **For protein expression** | |
| *ompR* | GCGGGATCCATGCAAGAGAATCACAAG/GCGAAGCTTTCATGCTTTATTGCCGTCCGG |
| *crp* | GCGGGATCCATGGTTCTCGGTAAGCCACAA/GCGAAGCTTTTAACGGGTGCCGTAAACGAC |
| **For LacZ fusion** | |
| *ompR* | GCGGAATTCGAAGTGCTGAAAATTGTTGACC/GCGGGATCCACGTAGACGCATATCGTCATC |
| *crp* | GCGGAATTCTGCCTTATCACCCGAATTTC/GCGAAGCTTTGCCTTATCACCCGAATTTC |
| *ompC* | GCGGAATTCTTGAAGTATGACGGGTATAACG/GCGGGATCCCACTGCCTGCAACCAATAAG |
| *ompF* | GCGGAATTCTACGGCACTGTACCGCATTC/GCGGGATCCGGCTAACAGAGCTGGGATTAC |
| *ompX* | GCGGAATTCGAGACAACGACATCCTTTGGAG/GCGGGATCCCTTTGACCTGCAAAGGCAGA |
| **For DNase I footprinting** | |
| *ompC* | GTGTTAGCCACAATGATAGG/CCATATAGAAAATGCCACC |
| *ompF* | CGTTCCCACGCACACC/GCAGTGTTCCATTCACAGACC |
| *ompX* | CCAGTGTAAATGAAATGCC/ACACCGTGCGACTATAGC |
| *ompR* | GAAGTGCTGAAAATTGTTGACC/ACGTAGACGCATATCGTCATC |
| *crp* | TGCCTTATCACCCGAATTTC/GCAATGAGACAGGAACCATTC |
| **For primer extension** | |
| *ompF* | GGCTAACAGAGCTGGGATTAC |
| *ompX* | CTAACACACACGCTGCTACC |
| *ompR* | ACGTAGACGCATATCGTCATC |
