# Supplementary material for: Regulatory effects of cAMP receptor protein (CRP) on porin genes and its own gene in Yersinia pestis
Source: BMC Microbiol. 2011 Feb 23;11:40. doi: 10.1186/1471-2180-11-40 (PMC3050693; doi:10.1186/1471-2180-11-40)
Supplement: Additional file 2 — Promoter activity of ompF within WT, Δcrp and C-crp. [file 1471-2180-11-40-S2.DOC]

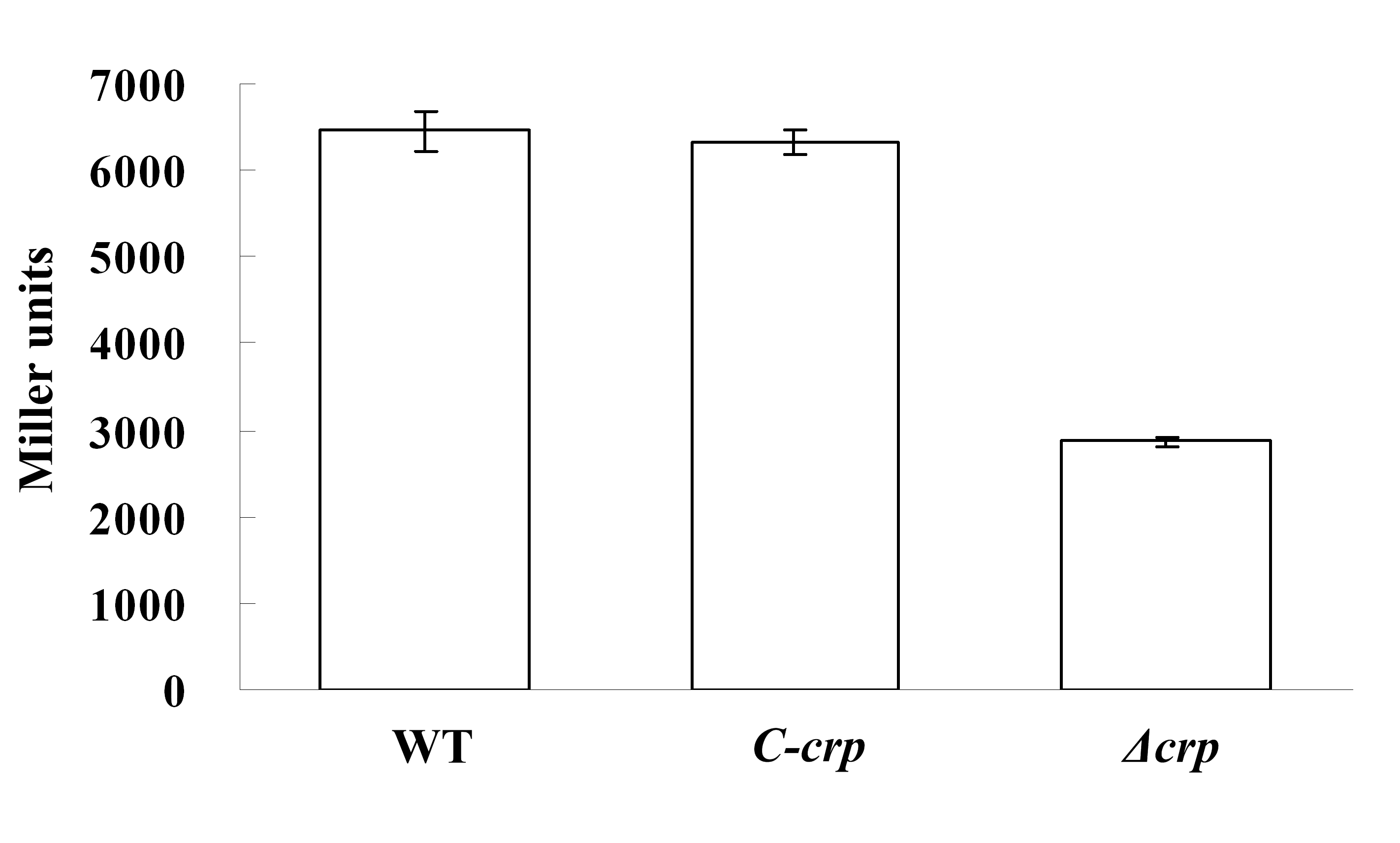


A promoter-proximal region of *ompF* was cloned into pRW50 containing a promotorless *lacZ* reporter gene, and transformed into WT, *Δcrp* (the *crp* null mutant) and *C-crp* (the complemented mutant) with the addition of 1 mM cAMP, respectively, to determine the promoter activity (β-Galactosidase activity in cellular extracts). The empty plasmid was also introduced into each strain as negative control, which gave extremely low promoter activity (data not shown).

Shown in the figure was the mean β-Galactosidase activity (Miller units) in WT, *Δcrp* and *C-crp*, respectively, subtracted by the corresponding negative control. The *ompF* gene was positively regulated by CRP as determined by several distinct methods (see text). As expected herein, the om*pF* promoter activity decreased significantly in *Δcrp* relative to both WT and *C-crp* (*P*<0.01), but gave no difference between WT and *C-crp* (*P*>0.05), which confirmed that the *crp* mutation was nonpolar.
